# Optimizing Crop Water Use for Drought and Climate Change Adaptation Requires a Multi-Scale Approach

**DOI:** 10.3389/fpls.2022.824720

**Published:** 2022-04-29

**Authors:** James D. Burridge, Alexandre Grondin, Vincent Vadez

**Affiliations:** ^1^DIADE Group, Cereal Root Systems, Institute de Recherche pour le Développement/Université de Montpellier, Montpellier, France; ^2^Adaptation des Plantes et Microorganismes Associés aux Stress Environnementaux, Laboratoire Mixte International, Dakar, Senegal; ^3^Centre d’Étude Régional pour l’Amélioration de l’Adaptation à la Sécheresse, Thiès, Senegal; ^4^International Crops Research Institute for Semi-Arid Tropics (ICRISAT), Patancheru, India

**Keywords:** drought, cross-scale coordination, water acquisition and use, selection criteria, transpiration restriction, vapor pressure deficit

## Abstract

Selection criteria that co-optimize water use efficiency and yield are needed to promote plant productivity in increasingly challenging and variable drought scenarios, particularly dryland cereals in the semi-arid tropics. Optimizing water use efficiency and yield fundamentally involves transpiration dynamics, where restriction of maximum transpiration rate helps to avoid early crop failure, while maximizing grain filling. Transpiration restriction can be regulated by multiple mechanisms and involves cross-organ coordination. This coordination involves complex feedbacks and feedforwards over time scales ranging from minutes to weeks, and from spatial scales ranging from cell membrane to crop canopy. Aquaporins have direct effect but various compensation and coordination pathways involve phenology, relative root and shoot growth, shoot architecture, root length distribution profile, as well as other architectural and anatomical aspects of plant form and function. We propose gravimetric phenotyping as an integrative, cross-scale solution to understand the dynamic, interwoven, and context-dependent coordination of transpiration regulation. The most fruitful breeding strategy is likely to be that which maintains focus on the phene of interest, namely, daily and season level transpiration dynamics. This direct selection approach is more precise than yield-based selection but sufficiently integrative to capture attenuating and complementary factors.

## Introduction

Increasing temperature, aridity, and unpredictability of rainfall events motivates the development of dryland cereal crops that produce grain in severe and variable drought scenarios, but still have high yield potential in less stressful scenarios. In these agroecological zones, high temperature, and low relative humidity can combine to make extremely taxing vapor pressure deficit (VPD) conditions, meaning more water transpired per carbon gained. High VPD conditions are predicted to become more common and more severe (Grossiord et al., 2020).

Root system architectural and anatomical traits or phenes (phene is to phenome as gene is to genome, thus phenotype is composed of phenes) that optimize water acquisition per unit carbon invested (Lynch, 2007, 2019) and “right-size” plant water usage (Borrell et al., 2014a; Lynch, 2018) are a positive step. Identifying and selecting for root trait plasticity may also be a useful step (Topp, 2016; Schneider and Lynch, 2020). Similarly, the ratio of shoot to root area is of fundamental importance for plant water balance (Hsiao and Acevedo, 1974). However, optimized root to shoot growth can have limited utility when there is simply a limited amount of soil water available.

In these scenarios, a strategy based upon parsimonious water usage co-optimizes transpiration, carbon fixation, and yield by conserving soil water for the grain filling stage (Richards et al., 2002; Zaman-Allah et al., 2011; Vadez et al., 2013a; Borrell et al., 2014b; Vadez, 2014; Hammer et al., 2020). Conserving soil water for grain filling can be achieved by limiting leaf area, limiting transpiration rate, or accelerating senescence of older leaves (Borrell et al., 2014a; George-Jaeggli et al., 2017; Sinclair et al., 2017). However, these adaptations may entail reduced yield potential under less stressful conditions (Gao et al., 2020a). Constraining daily transpiration rates from climbing above a certain threshold, when VPD is high (i.e., when the trade-offs between carbon fixation and water loss becomes too costly), is promising means to conserve water for the grain filling period, without reducing total leaf (Sinclair et al., 2017).

Inducible limitation of maximum transpiration increases transpiration efficiency saves water over the course of a day and over the course of the season (Sinclair et al., 2005, 2017). Modeling studies have shown the great benefit with little trade-off of high VPD induced transpiration restriction in soybean (Sinclair et al., 2010), maize (Messina et al., 2015), and sorghum (Sinclair and Muchow, 2001; Kholová et al., 2014). However, there may be trade-offs between leaf cooling and transpiration restriction under very high temperatures. Field studies indicate that transpiration restriction is related to greater yield for maize, sorghum, pearl millet, and wheat under severe terminal drought conditions (Sinclair et al., 2017; Tharanya et al., 2018a; Medina et al., 2019). Selection for transpiration restriction phenotypes has been implemented in peanut, maize, and soybean breeding programs and cultivars have been generated exhibiting soil water conservation strategies (Shekoofa and Sinclair, 2018). Similar transpiration restriction strategies may conserve soil water and increase dryland production of other annual crops (Belko et al., 2012; Polania et al., 2016). Understanding species and genotype level variation in transpiration restriction may help accelerate crop genetic improvement.

## Part 1: Regulation of Transpiration Restriction by Plant Hydraulics

**Figure 1:**
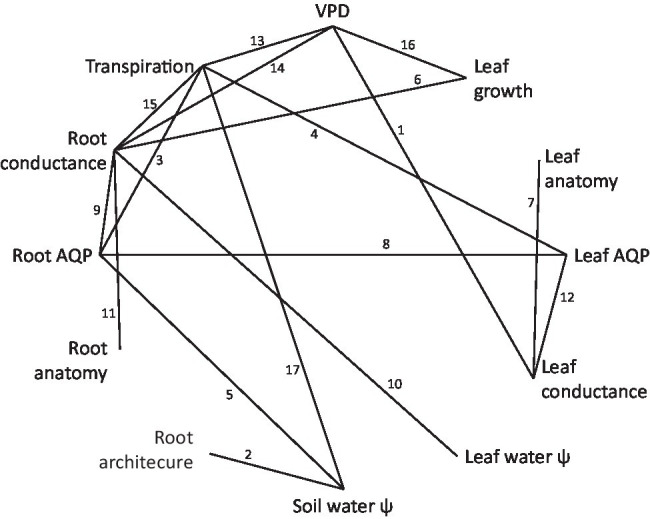
Non-hierarchical arrangement of actors and processes involved in plant water acquisition, water transport, and transpiration regulation across all levels of plant organization. Numbered lines between circled actors correspond to publications demonstrating indicated connection, listed in Table 1. The network is not intended to be exhaustively populated, but rather representative, and indicates a high degree of interconnectivity, yet with substantial lacunae among actors and processes that are logically related. It suggests that as we accumulate more data, we find more interactions and more complexity.

**Figure 2:**
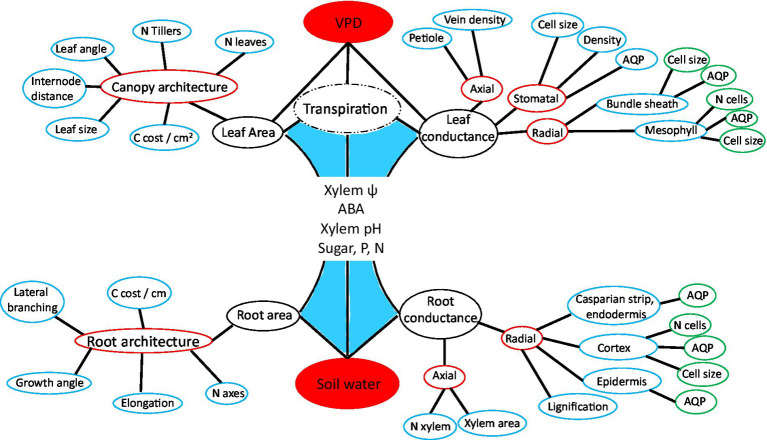
Hierarchically structured network diagram of phenes influencing plant water acquisition and transport, including the same actors and processes as in Figure 1. This projection of plant hydraulic regulation highlights the distal position of aquaporins in relation to tissue level conductance, the complementary role of relative shoot and root growth, and the nested structure of plant form and function. C is an abbreviation for carbon and *N* for number.

Plants connect the pedosphere, with relatively high water potential, to the atmosphere, with relatively low water potential. Water movement along this soil–plant–air continuum is driven by a water potential gradient, as described by Ohm’s law and the cohesion–tension theory (Tyree, 1997; Carminati and Javaux, 2020). Plants use a network of specialized architectural, anatomical, morphological, and functional mechanisms to regulate the axial and radial flow of water (Steudle, 2000). Root radial water transport involves passage through the epidermis, cortex, endodermis, and xylem parenchyma *via* the symplastic (cell to cell) or apoplasticaly (through cell walls and intercellular spaces; McCully and Canny, 1988; Bramley et al., 2007). Water ascends axially by tension and cohesion through root, stem, petiole, and leaf vein xylem vessels. Tension draws water from the leaf veins, across multiple sets of cell membranes, including the bundle sheath, mesophyll, or epidermal cells. Water vapor then diffuses through the cuticle, or in a highly controlled fashion through the stomatal cavity. The actors and processes involved in hydraulic regulation are presented using a non-structured, conceptual arrangement in Figure 1, which serves to guide the literature review. Supporting information is supplied in Table 1; Supplementary Table 1. Functionally structured perspectives of plant hydraulic regulation are provided in Figure 2; Supplementary Figure 1.

**Table 1:** List of publications demonstrating links between nodes.

Edge	Node 1	Node 2	Reference numbers
1	VPD	Leaf conductance	1, 18
2	Soil water potential	RSA	2, 3, 40
3	Root AQP	Transpiration	4, 5, 6, 20, 22, 25, 26, 31, 32
4	Leaf AQP	Transpiration	7, 18, 19, 21, 27, 28, 33
5	Soil water potential	Root AQP	8, 9
6	Leaf growth	Root conductance	10, 11, 22, 25, 31, 32
7	Leaf anatomy	Leaf conductance	7, 13, 14, 30, 37, 41, 42
8	Root AQP	Leaf AQP	8
9	Root AQP	Root conductance	15, 16, 17, 23, 24, 25, 31, 32, 34, 38, 39
10	Root conductance	Leaf water potential	22
11	Root anatomy	Root conductance	12, 16, 23, 24
12	Leaf conductance	Leaf AQP	29, 33, 35, 36, 38
13	VPD	Transpiration	43, 44, 45, 46, 47, 49, 50, 51, 52
14	VPD	Root conductance	44, 48
15	Root conductance	Transpiration	44, 48
16	VPD	Leaf growth	49, 53
17	Soil water potential	Transpiration	52, 54, 55, 56

Edge numbers correspond to links between nodes in Figure 1. References and reference numbers presented in Supplementary Table 1.

### Root Conductance

Root-based regulation of transpiration can be divided into radial and axial conductance. Root axial water conductance is typically not considered the most rate limiting step, but genotypic differences do exist in xylem number and diameter, which determine axial conductance capacity, and can relate to transpiration dynamics and adaptation to drought stress (Prince et al., 2017; Nogueira et al., 2020; Strock et al., 2020). Reduced seminal root xylem conductance capacity was the basis of developing wheat cultivars adapted to the water-limited Australian context (Richards and Passioura, 1989). The utility of reduced root axial conductance capacity for late season soil water conservation in wheat has been further supported in recent work (Hendel et al., 2021). There may also be the possibility for longitudinal adjustments of xylem conduits (Meunier et al., 2017), regulation at the root to shoot junction (Meunier et al., 2018), as well as among the various attributes of protoxylem and metaxylem vessels, their pits (Xu et al., 2020) or perforation plates (Gao et al., 2020b). These may be part of a suite of embolism response traits and do not preclude the possibility that embolism is itself a means of restricting transpiration (McCully, 1999), in which aquaporins play a key recovery role (Secchi et al., 2017).

Root anatomical phenes related to radial water transport include distance between root tip and suberized zone, as well as completeness of suberization (Barberon et al., 2016; Doblas et al., 2017) and lignification (Foster and Miklavcic, 2017). Construction of Casparian bands, suberin lamellae, and lignification may respond dynamically to abiotic stress factors and be deployed differentially on roots of different diameters and class (Tylová et al., 2017). Variation in cortex cell file number, or cortex cell size may lead to a different proportion of cell-to-intercellular spaces, which are hypothesized to affect root hydraulics (Vadez, 2014), with some evidence in pearl millet (Kholová et al., 2016). Genotypic variation in radial conductance pathways has been observed in chickpea (Sivasakthi et al., 2017, 2020). Similar effects of anatomical differences in the root radial water transport pathway were observed in wheat and lupin, showing predominance of apoplast water transport in lupin, whereas wheat dependent mostly on Hg-sensitive aquaporin in the endodermis (Bramley et al., 2007).

Root radial conductance is influenced by AQP at various membranes including; epidermis, outer cortex (Ranathunge et al., 2004), endodermis, and Casparian strip (Grondin et al., 2016). AQP expression in rice shoots and roots suggests AQP mediated root conductance was most limiting to mid-day transpiration (Nada and Abogadallah, 2014). A pearl millet aquaporin gene transferred to tobacco conferred greater drought, heat, and higher water use efficiency (Reddy et al., 2022). Knockout and overexpression mutants showed a specific AQP isoform in maize roots was an important regulator of root hydraulic conductance, with effects on plant growth (Ding et al., 2020). The same study suggested non-uniform patterns of radial conductance, implying aquaporin function must integrate with root anatomy.

### Leaf Conductance

Leaf conductance is an aggregate phene, *sensu* (York et al., 2013), integrating leaf vein anatomy, stomatal density size, and aperture, as well as xylem parenchyma, bundle sheath, and mesophyll cell number, size, and density, in addition to AQP function (Sack and Holbrook, 2006). This presents a variety of regulatory opportunities operating at different scales, involving different actors and signaling pathways. Outside xylem conductance, meaning conductance on the path between xylem and sites of evaporation (Scoffoni et al., 2017; Corso et al., 2020) contributes to transpiration restriction (Sinclair et al., 2008). Guard cell conductance is involved in transpiration regulation in response to VPD (Sinclair et al., 2008). Regulation of conductance by bundle sheath cells, likely attributable to AQP, was demonstrated using applied ABA and mercury (Shatil-Cohen et al., 2011). Subsequent work used microRNA AQP silencing to demonstrate a role of AQP at the bundle sheath to mesophyll transition (Sade et al., 2014, 2015). Knockout mutants were used to demonstrate that light-dependent activity of a single AQP isoform in leaf veins is a major regulator of leaf conductance (Prado et al., 2013).

Anatomy, and its interaction with membrane level conductance, may play a role in regulating transpiration dynamics. Within the leaf, two-thirds of outside xylem hydraulic conductance was attributed to vapor transport, which is strongly influenced by distance between veins, distance between vein terminus and stomata, as well as spongy mesophyll anatomy (Sack and Frole, 2006; Sack and Holbrook, 2006; Brodribb et al., 2007; Sack and Scoffoni, 2013; Buckley et al., 2015). One study noted various leaf anatomic factors involved in transpiration efficiency and observed distinct association of anatomy and aquaporin function at different drought intensities (Henry et al., 2019). Transpiration restriction phenotypes were associated with modified epidermal cell size and stomatal density in response to VPD in cotton (Devi and Reddy, 2018). Leaf petiole conductance, and by implication, all of xylem axial conductance could be involved in hydraulic regulation (Postaire et al., 2010). The integration of these water transport factors into a complex series has potential regulatory ability in addition to the regulation of individual components (Zwieniecki et al., 2007). Indeed, canopy development, leaf anatomy, root growth, and water uptake have been related to the stay-green phenotype (Borrell et al., 2014a), which has transpiration restriction as an underlying phenotype. We conclude that focus is needed on the interactions among steps of the water transport pathway, as well as interactions with anatomy, irradiance, leaf water status, and growth to fully understand the regulation of leaf hydraulics (Prado and Maurel, 2013).

### Cross-Organ Environmental Responses

Highly dynamic root and leaf expression of multiple AQP was related to maintenance of water use efficiency over the course of a day in sorghum but not in maize (Hasan et al., 2017). Maize, sorghum, and pearl millet may deploy transpiration restriction strategies along a spectrum of reduced leaf area expansion rate or restricted transpiration rate (Sinclair et al., 2017; Choudhary et al., 2020). These species may also vary in their transpiration restriction across different soils (Vadez et al., 2021). At high VPD maize restricted maximum transpiration rate, and transpiration rate became more sensitive to soil drying, while pearl millet and sorghum relied mainly on reduced leaf expansion as a means to reduce transpiration (Choudhary et al., 2020). Genotypic variation also exists for the ability to restrict transpiration rate in response to environmental cues, such as high VPD or highly negative soil matric potential (Choudhary and Sinclair, 2014; Sinclair et al., 2017; Medina et al., 2019). Measurements of leaf and whole plant hydraulic conductance in 12 maize genotypes suggest coordination between root and shoot conductance effectively regulated transpiration in response to high VPD (Sunita et al., 2014). However, work using 20 sorghum genotypes found genotypic variation in both leaf and root conductance and suggested a shoot-based causal mechanism of limited maximum transpiration rate (Choudhary and Sinclair, 2014). Growth chamber, glass house, and field experiments on sorghum linked genetic variation for response of maximum, transpiration rate under increasing VDP to water saving, but observed significantly different results between experiments (Karthika et al., 2019). In sum, the data suggest there are different transpiration restriction strategies and mechanisms with substantial species and genotypic level variation, some of which is environmentally dependent.

## Part 2: Transpiration Restriction Can Involve Multiple Signals, Hysteresis, and Phenotypic Integration

Transpiration is a dynamic process, involving coordination of structural and functional aspects across organizational scales in both roots and shoots. Therefore, identifying transpiration restriction mechanisms can be very complex (Figure 2; Supplementary Figure 1). Outcomes of studies on transpiration restriction are influenced by plant size (Sadok and Sinclair, 2010), time interval studied (Tardieu and Parent, 2017), timing of water stress (Shekoofa and Sinclair, 2018), stage of water stress (Pou et al., 2013), severity of stress (Lovisolo et al., 2010), temperature (Yang et al., 2012), and breeding history (Vadez et al., 2011). Transpiration restriction can also be influenced by employing an isohydric or anisohydric strategy (Vandeleur et al., 2009), and generally if plants are “water savers” or “water spenders.”

A water spender, or profligate water use strategy, is associated with large leaf area and/or unrestricted transpiration in response to high VPD. A water saver, or conservative water use strategy, would reduce leaf area and/or restrict transpiration at high VPD. Isohydric behavior entails sensitive stomatal control that maintains relatively constant leaf water potential, even when soil water is limited or VPD is high. An anisohyric strategy would tolerate a drop in leaf water potential (Grossiord et al., 2020). Genotypes with a water saver strategy may rely more on the apoplastic pathway, whereas water spenders may rely more on the symplastic pathway, but pathway utilization can also depend on growth rate (Grondin et al., 2020; Sivasakthi et al., 2020).

Practices, such as deficit irrigation (Chaves et al., 2010) and cropping system (Sadras et al., 1989), can influence if a plant employs an isohydric or anisohydric strategy. However, these are just two points on a spectrum and strategy can vary by genotype and over time within a single plant (Knipfer et al., 2020). This flexibility along the iso- to anisohydric continuum has been characterized using the hydroscape concept, defined as the area between predawn and mid-day plant water potential regression lines, which captures processes across the soil–plant–atmosphere pathway (Meinzer et al., 2016; Javaux and Carminati, 2021). We conclude that a more robust, yet accurate, selection for transpiration restriction involves the cross-scale dynamic coordination of a spatially and temporally complex set of interacting phenes and processes.

### Multiple Types of Signals Can Influence Membrane Conductance

Coordination of root and shoot AQP expression, localization, and function makes use of multiple signals, such as ABA, xylem pH (Davies et al., 2002), and xylem pressure potential itself (Chaumont and Tyerman, 2014; Vandeleur et al., 2014) and may require the integration of multiple signals (Comstock, 2002). Multiple lines of research suggest the importance of a hydraulic signal but differ as to if that signal originates from root or shoot (Fuchs and Livingston, 1996; Yao et al., 2001; Vandeleur et al., 2014). Soil hydraulic conductivity, referring to the hydraulic connection between roots and the soil, has recently been identified as an important signal and regulator of plant hydraulics, transpiration, and stomatal response to drought (Carminati and Javaux, 2020; Hayat et al., 2020; Cai et al., 2021). The rapidity of transpiration response to VPD suggests hydraulic rather than biochemical signals are the immediate mechanisms (Kholová et al., 2010b). However, there is evidence that hydraulic and biochemical signals interact, perhaps over longer time scales (Christmann et al., 2013).

Similarly, partial root drying studies suggest root originating signals that do not involve AQP transcription (Li et al., 2008). For example, ABA and xylem pH can influence transpiration (Davies et al., 2002). The role of ABA as both a local and long-distance signal of soil water limitation has been identified in several species (Dodd, 2005; Wang et al., 2019), although a role for cytokinin has also been suggested (Kudoyarova et al., 2007). Other work suggests ABA signaling operates in conjunction with hydraulic signals, which in turn affects hydraulic conductance of bundle sheath cells (Sade et al., 2014) and may promote root growth, all while being sensitive to stress severity (Miao et al., 2021). Other research suggesting both root and shoot need to be in communication (Castro et al., 2019) are consistent with the multiple signal hypotheses. ABA accumulation in the root has been linked to increased root hydraulic conductivity (Sharipova et al., 2016). However, enhanced root ABA production was linked to reduced leaf conductance under non-limiting conditions, and greater transpiration restriction under high VPD (Thompson et al., 2007).

In summary, particular signaling mechanisms have demonstratable involvement in communicating and responding to particular environmental conditions in particular experimental systems. Studies on membrane or organ level conductance usually involve transgenic, pharmacological, stem girdling, or de-topping approaches that have distinct limitations. These types of studies may fail to account for compensatory mechanisms at other organs and scales, like the opposite effects of ABA on leaf and root conductance described above (Thompson et al., 2007; Sharipova et al., 2016). Furthermore, there appears to be little consistency in signals identified as mechanisms of transpiration regulation across experimental systems. This suggests a high degree of environmental dependency and implies that the actors, forces, and signals identified may not be commensurate with an unperturbed system. For these reasons reductionist experimental systems are ill-suited to deciphering the complexity of the whole system (Tardieu and Parent, 2017) and a broader perspective is warranted.

### Hysteresis Influences Plant Responses

Hysteresis, in the context of a water acquisition and use, involves how the plant’s environment and history affects signal and response mechanisms. Hysteresis can thus describe a type of cross-scale legacy effect, involving previous architectural, anatomical, and cellular responses. Hysteresis also implies functional factors, such as stomatal aperture, water use strategy, and isohydricity, and if water is being absorbed into the root *via* symplastic or apoplastic pathways, which can make use of different AQP (Javot et al., 2003). Shifting between isohydric vs. anisohydric strategies (Sade et al., 2012) may depend upon a combination of soil moisture, VPD, and hormonal cues (Rogiers et al., 2012) interacting in a tissue-specific and dose-dependent manner (Rosales et al., 2019). The shift in strategies likely involves modified AQP expression (Sade et al., 2009), different root radial transport pathways (Tharanya et al., 2018b), as well as different signaling pathways in different scenarios (Aroca et al., 2012; Moshelion et al., 2015; Rosales et al., 2019). For example, Pou et al. (2013) found that the apoplastic pathway was more important during water stress. Furthermore, dynamic transpiration regulation, and its regulation by aquaporins, can depend upon N availability (Cramer et al., 2009; Di Pietro et al., 2013; Ding et al., 2018) and its degree and duration of deprivation (Dodd et al., 2003).

The existence of multiple overlapping regulation pathways is further shown with research in grapevine, suggesting not only that there is variation in water use strategy by cultivar, which has impact on WUE, and is dependent on type and severity of stress (Lovisolo et al., 2010), but also that the same cultivar can employ different strategies based on legacy effects (Chaves et al., 2010). Transpiration response to VPD may involve different mechanisms under different environmental scenarios (Pou et al., 2013; Sunita et al., 2014; Henry et al., 2019). A study comparing maize, sorghum, and pearl millet growth under contrasting VPD conditions, and then exposed to high VPD, showed species level variation in transpiration dynamics and leaf area, contingent upon growing conditions (Choudhary et al., 2020). Work in pearl millet found diurnal variation and VPD treatment dependency on AQP expression patterns among VPD sensitive and insensitive genotypes (Reddy et al., 2017). The impact of AQP overexpression in two rice cultivars on growth, transpiration patterns, and ultimately water use efficiency, was contingent upon root to shoot ratio and the expression of other aquaporins (Nada and Abogadallah, 2020). AQP downregulation can be balanced by increases in root size, bundle sheath cell osmotic permeability, and other mechanism (Kaldenhoff et al., 1998; Martre et al., 2002; Siefritz et al., 2002; Vandeleur et al., 2014). These examples demonstrate the existence of dynamic cross-scale compensation and a high degree of interconnectivity in transpiration regulation.

In terms of environmental interactions, temperature influences transient transpiration response to VPD (Seversike et al., 2013) and soil drying influences root morphology and transpiration response in soybean (Seversike et al., 2014). Genetic differences in root architecture and variation in root growth response to environmental factors may interact with transpiration regulation and have different impacts on transpiration, canopy temperature, and yield in different environments (Henry et al., 2011). Fully describing a signal—response pathway may require multiple theories, similar to how explanations of nutrient regulation of plant growth differ in accord with the limiting nutrient (Rubio et al., 2003). Indeed, it has been proposed that ABA signals originating from either root or shoot overlap with and mediate hydraulic signals to influence stomatal conductance and leaf hydraulic conductance (Pantin et al., 2013). The high level of interactivity among signals and environmental dependence suggests multiple signals operate in an integrated fashion to influence the emergent transpiration response phenotype. In summary, the legacy of previous physiological responses dictates available responses to the next set of conditions and needs to be taken into account when examining transient responses.

### Phenotypes Integrate Across Scales

The integration of water acquisition, transport, and daily and season level water use dynamics, along with phenology, influence the effectiveness of the plant water use strategy in a given environment. Temporal dynamics in water availability and use introduces the need for cross-scale coordination of processes, such as plastic root growth (Topp, 2016; Schneider and Lynch, 2020), involving both architectural (Schneider et al., 2020b) and anatomical (Schneider et al., 2020a) adjustments. Spatio-temporal variation in hydraulic conductance among different root classes and ages highlights an additional layer of variation (Schneider et al., 2020c). Root architecture, xylem characteristics, and stomatal conductance integrate as a coordinated network in maize to enhance performance (Gleason et al., 2019). Integrated root architectural, xylem conductance capacity and maturity group phenotypes have been related to performance and water use strategies in *Phaseolus* (Strock et al., 2020) and in *Zea mays* (York and Lynch, 2015; Klein et al., 2020). Integrated phenotypes involving root architecture, root hydraulic conductance capacity, and phenology have been hypothesized to exist in grain legumes (Burridge et al., 2020) and observed at the gene pool and race level in common bean (Jochua et al., 2020).

There are likely multiple mechanisms for transpiration optimization that are composed of distinct integrated phenotypes involving architectural, anatomical, cellular, and even soil and canopy elements integrating with growth, phenology, and transpiration patterns. For instance, decades of research on the slow wilting phenomenon in soybean have uncovered multiple mechanisms (Kunert and Vorster, 2020) including reduced stomatal conductance (Tanaka et al., 2010), contrasting leaf morphology (Hudak and Patterson, 1995), a larger, more fibrous root system (Pantalone et al., 1996) and by unknown mechanism(s) (Bagherzadi et al., 2017). A recent paper (Ye et al., 2020) used different soybean germplasm than a previous study and identified transpiration restriction mechanisms distinct from the previously identified silver sensitive mechanism (Sadok and Sinclair, 2010). These findings support early work suggesting multiple water conservation mechanisms in soybean (Charlson et al., 2009) and again suggest phenotypes integrate to coordinate transpiration, growth, and soil water use.

Integrated transpiration regulation phenotypes involving conductance, transpiration, canopy size, and phenology have also been observed in sorghum, wheat, chickpea, and pearl millet. A study of four stay-green QTL in sorghum found the four QTL regulated canopy size but also affected leaf anatomy, root growth, and water uptake (Borrell et al., 2014a). Contrasting integrated phenotypes, involving root axial and transmembrane conductance, could be involved in wheat drought tolerance strategies (Schoppach and Sadok, 2012; Schoppach et al., 2014). In chickpea, early vigor, as gauged by canopy size, was related to transpiration restriction and preferential use of the root apoplastic pathway (Sivasakthi et al., 2020). Similarly, greater propensity to restrict transpiration *via* root conductance was associated with larger canopy size in pearl millet (Kholová et al., 2010b; Tharanya et al., 2018b) and chickpea (Zaman-Allah et al., 2011) suggesting transpiration regulation mechanisms specific for large or small canopy size.

Root hairs provide another example for how phenotypic integration connects to the issue of coordination between root and leaf conductance. In addition to xylem embolisms, hydraulic disruptions between root and soil (Newman, 1969; Draye et al., 2010; Carminati and Javaux, 2020; Hayat et al., 2020) may be another type of hydraulic signal, which is theoretically impacted by heterogenous soil conductivity and particle size (von Jeetze et al., 2020). Recent evidence demonstrates that roots signal this localized hydraulic resistance, which in turn triggers stomatal closure before leaf conductivity reduces (Rodriguez-Dominguez and Brodribb, 2020). Apart from potentially being involved in this signaling, root hairs may help maintain rhizosphere to bulk soil connectivity (Segal et al., 2008; Draye et al., 2010; Lobet et al., 2014; Carminati et al., 2017). Root hair length and density may thus integrate with root length distribution profile and daily transpiration dynamics to promote increased season level transpiration (Tardieu and Parent, 2017).

## Part 3: Toward Effective Integrative Phenotyping

There seems to be consensus in the literature that the primary short-term mechanisms for fine-tuning transpiration to environment and plant needs involve aquaporins. Aquaporins regulate conductance at the membrane level in both root and leaf (Hachez et al., 2006a, 2008, 2012; Wang et al., 2019). For that reason, selection for particular AQP isoforms or AQP expression levels are tempting targets for engineering transpiration efficiency, even while intricacies of AQP function are acknowledged (Hachez et al., 2006b; Afzal et al., 2016; Zargar et al., 2017). One of the challenges is that there is no consensus on a correlation between AQP abundance and tissue level conductivity (Aroca et al., 2012). The challenges these intricacies pose for genetic improvement are further indicated by how different research programs, using different experimental designs and species, have contrastingly attributed hydraulic regulation almost exclusively to the root (Rodriguez-Dominguez and Brodribb, 2020) or the shoot (Sinclair et al., 2008; Figure 1; Table 1). Delving into the many studies on aquaporins makes clear only that there is extensive interaction, compensation, and redundancy among aquaporins within and across scales, across organs, as well as architectural and anatomical effects. We therefore conclude that AQP are currently ill-suited to be used as a selection criterion for the genetic improvement of transpiration responses to environmental conditions.

Identification of robust selection criteria, with good heritability, becomes complicated when phenotypes are complex, cross-scale, as well as legacy and environmentally dependent. Inducible transpiration restriction is one such multi-scale phenotype. It requires the coordination of plant water acquisition, transport, growth, and transpiration and is regulated by multiple actors and pathways. These actors and pathways can vary according to type, severity, and timing of stress, and in relation to plant size, phenology, and hysteresis. Viewing the dynamic coordination of plant transpiration and growth from this perspective highlights three potential approaches to accelerate crop genetic improvement. Firstly, multi-scale modeling and machine learning could be used to predict outcomes and limit the number of phenotypic combinations to test empirically. Secondly, there is a potentially indicative phene. Thirdly, we propose an integrative direct selection strategy.

### Multi-Scale Models

Understanding how modifications of transpiration and growth feedback and feedforward with tissue hydraulic conductance, stomatal conductance, shoot, and root architecture, hormones, and aquaporins is critical for identifying selection criteria for inducible transpiration restriction phenotypes (Tardieu and Parent, 2017). Multiple recent calls for integrating multi-scale computation models with crops simulations emphasize the need to integrate across spatial and temporal scales (Chew et al., 2017; Marshall-Colon et al., 2017; Benes et al., 2020; Peng et al., 2020), across disciplines (Hammer, 2020) and even beyond the plant and into the rhizosphere and soil (Lobet et al., 2014).

Organizing soil, plant, and canopy simulation models in nested networks, linked in multiple ways mirrors the function of the inducible transpiration restriction phenotype. Developing and benchmarking multi-scale models offers the potential to apply machine learning to data generated by said models. While truly multi-scale models are only just emerging (Ajmera et al., 2022), and benchmarking has much progress to make (Schnepf et al., 2020), machine learning could conceivably help identify latent features and highlight selection targets. Models may help decipher how modifying a particular phene integrates with other phenes and effects the emergent phenotype of yield, in a given environment. A yield-risk approach (Hammer et al., 2020) could then be applied to evaluate the influence of the timing, sensitivity, and degree of changes in transpiration. Emergent phenotypes related to transpiration optimization could then be directly selected for using traditional breeding techniques.

### Xylem Conductance Capacity May Indicate Transpiration Strategy

Elementary plant phenes may indicate broader strategies, similarly to how selection for genes that lie at the hubs of gene networks likely modulate more complex phenotypes than genes at the outer spokes of a network (Dietz et al., 2010). Root axial conductance capacity, as estimated by xylem vessel number and diameter using the Hagen–Poiseuille equation, is an example (Tyree et al., 1994). Xylem conductance capacity can be estimated using laser ablation tomography and has been linked to performance (Nogueira et al., 2020; Schneider et al., 2020a; Strock et al., 2020; Hendel et al., 2021). Potentially further facilitating selection, is the observation that xylem conductance phenotypes of young plants were related to mature plant phenotype (Falk et al., 2020). It should be noted here that the targeted phenotype, that is, xylem conductance, would be an estimate, which could overestimate the actual value. Root anatomical modifications, such as suberization and lignin deposition, are also identifiable using laser ablation tomography (Strock et al., 2019) and lignification may have added benefits related to soil resistivity as well as pathogen and root pest resistance (Schneider et al., 2021). AQP may integrate with xylem parenchyma traits to refill xylem embolisms (Secchi et al., 2017), which laser ablation tomography could help address by quantifying parenchyma number, size, and positioning.

Xylem conductance capacity could indicate water use strategy for two reasons. Firstly, under-utilizing a high conductance capacity xylem phenotype would unnecessarily increase the risk of cavitation. Empirical and modeling evidence suggests that plants operate near the upper threshold of xylem imposed limits on hydraulic conductance (Sperry et al., 1998). Conceptually this makes sense, to avoid cavitation risk and to not waste the construction and maintenance costs of xylem and parenchyma. Secondly, elevated transpiration rates could not occur with a low xylem conductance phenotype, precluding the possibility for high transpiration, photosynthesis, and growth rates. Selection for reduced xylem conductance capacity estimated using root anatomical cross sections may thus be an easy way to select for reduced transpiration rate. Alternatively, a high conductance capacity phenotype may indicate a risk-taking approach involving highly dynamic transpiration regulation imposed at the cell membrane scale.

### Direct Selection for Transpiration Restriction

Direct phenotypic selection of inducible transpiration restriction in the target environment using realistic systems, such as gravimetric phenotyping, overcomes the potentially confounding cross-scale interactions and compensatory mechanisms to which organ level or controlled environment studies are sensitive. This type of direct phenotypic selection targets the emergent, or integrated phenotype, rather than lower-level component phenes, and acknowledges that feedbacks and compensation among component processes can obscure plant level processes and the ultimate target, yield (Vadez et al., 2013a; Kholová et al., 2016).

Phenotyping transpiration dynamics in a field-based lysimeter system, with realistic VPD and progressive soil drying captures the aggregate phenotype of interest as well as component phenes (Vadez et al., 2015; Kar et al., 2020, 2021). While field-based lysimeters have significant construction and operating costs, they have demonstrated utility for both trait-based and QTL-based selection (Kholová et al., 2012; Karthika et al., 2019). Heritability values for metrics describing transpiration dynamics range from moderate to high (Aparna et al., 2015; Sivasakthi et al., 2018; Tharanya et al., 2018a). This type of system uses lysimeters of large enough depth and total volume that permits additional root exploration, but with density similar to farmer’s conditions. It facilitates direct phenotypic selection of transpiration restriction, particular transpiration strategies and transpiration efficiency without eliminating dynamic and interacting environmental and plant factors (Kholová et al., 2012; Vadez et al., 2013b; Tharanya et al., 2018a,b). Weekly weighings are adequate to identify genotypes that employ early season water conservation and enable late season transpiration and grain filling (Vadez et al., 2013a; Tharanya et al., 2018a). However, weekly weights do not necessarily permit distinguishing if the mechanisms of water saving arises from leaf area dynamics, daily transpiration dynamics, or weekly transpiration dynamics.

Significant insight on daily dynamics, and in particular transpiration restriction in response to daily VPD (Ryan et al., 2016), can be gleaned from three (Kholová et al., 2012) or even one daily measurement (Choudhary et al., 2020). A similar system enables minute level resolution transpiration measurements and permits selection for amplitude of daily transpiration restriction (Vadez et al., 2015; Sivasakthi et al., 2018). Studying daily transpiration dynamics under variable VPD, as well as under progressive soil drying (Karthika et al., 2019), may reveal multiple useful transpiration patterns (Kholová et al., 2016). Non-destructive shoot imaging enables quantification of leaf area dynamics. Root systems of smaller plants can be washed and measured to reveal differences in root system size and root to shoot ratio. Combined utilization of these lysimeter systems capture hourly, daily, and season level interactions between soil water acquisition and use. These systems can quantify feedbacks among root investment, leaf area development, phenology, and density. By imposing realistic environmental conditions and enabling complex feedbacks to impact performance, gravimetric phenotyping offers the chance to identify superior integrated phenotypes and accelerate genetic improvement.

### Next Challenges

Of primary importance for selecting for resilience to current and future climates is addressing the utility of favorable transpiration dynamics in progressive soil drying scenarios. Enhanced resilience to terminal drought likely involves multi-scale coordination of water acquisition and use. Feedbacks among environment and phenes including axial root and leaf growth dynamics, tiller initiation, and transpiration dynamics quickly become complex and result in many trait combinations. The question of tillering, which relates to canopy density, leads to another very interesting set of questions involving if increasing planting density may reduce soil evaporation and create a favorable in-canopy micro-climate that improves the water loss to carbon gain ratio. In short, the challenge is to develop the conceptual frameworks, phenotyping platforms, and models that integrate across scales and capture overarching meta-mechanisms, such as inducible transpiration restriction, in order to identify important and selectable phenes.

## Conclusion

Our ultimate goal is the identification of robust selection criteria for water acquisition and use optimization, likely including inducible transpiration restriction. These selection criteria should optimize yield in increasingly variable high-temperature and drought-prone environments. A review of the literature suggests that transpiration restriction can lead to an optimized transpiration phenotype through multiple mechanisms and that multiple coordination pathways may be involved. Pharmacological or gene editing tools, when used in isolation, are poorly positioned to detect dynamic, hysteretic, multi-element, and multi-scale coordination associated with overlapping transpiration regulation pathways. Directly phenotyping for transpiration restriction in response to high VPD or limited soil water has demonstrated its utility for QTL and trait-based selection. Efforts to increase drought tolerance *via* the optimization of water acquisition and transpiration should focus on daily and season level transpiration dynamics at the whole plant level. This direct selection approach is likely to identify key integrated phenotypes and coordination mechanisms that have immediate utility for a breeding pipeline.

## Author Contributions

JB, AG, and VV conceived the review. VV secured the funding. JB wrote the review with comments and edits from AG and VV. All authors contributed to the article and approved the submitted version.

## Funding

The paper was written and supported under the Make Our Planet Great Again (MOPGA) ICARUS project (Improve Crops in Arid Regions and Future Climates) funded by the Agence Nationale de la Recherche (ANR, grant ANR-17-MPGA-0011).

## Conflict of Interest

The authors declare that the research was conducted in the absence of any commercial or financial relationships that could be construed as a potential conflict of interest.

## Publisher’s Note

All claims expressed in this article are solely those of the authors and do not necessarily represent those of their affiliated organizations, or those of the publisher, the editors and the reviewers. Any product that may be evaluated in this article, or claim that may be made by its manufacturer, is not guaranteed or endorsed by the publisher.

## Supplementary Material

The Supplementary Material for this article can be found online at: https://www.frontiersin.org/articles/10.3389/fpls.2022.824720/full#supplementary-material

Supplementary Figure 1 Functional projection of plant hydraulic regulation foregrounding nested structural hierarchies associated with the four general mechanisms (root and shoot architecture and conductance) governing transpiration and growth. Solid arrows depict biochemical and hydraulic signaling mechanisms within and between structural hierarchies and include transmembrane pressure potential, xylem pressure potential, pH, hormones, as well as carbohydrate, and nutrient concentration. Dashed arrows indicate interactions with environmental factors. Dotted arrows indicate interactions with phenology and planting density.
