# Supplementary figures and images for: Optimizing Crop Water Use for Drought and Climate Change Adaptation Requires a Multi-Scale Approach
Source: Front Plant Sci. 2022 Apr 29;13:824720. doi: 10.3389/fpls.2022.824720 (PMC9100818; doi:10.3389/fpls.2022.824720)

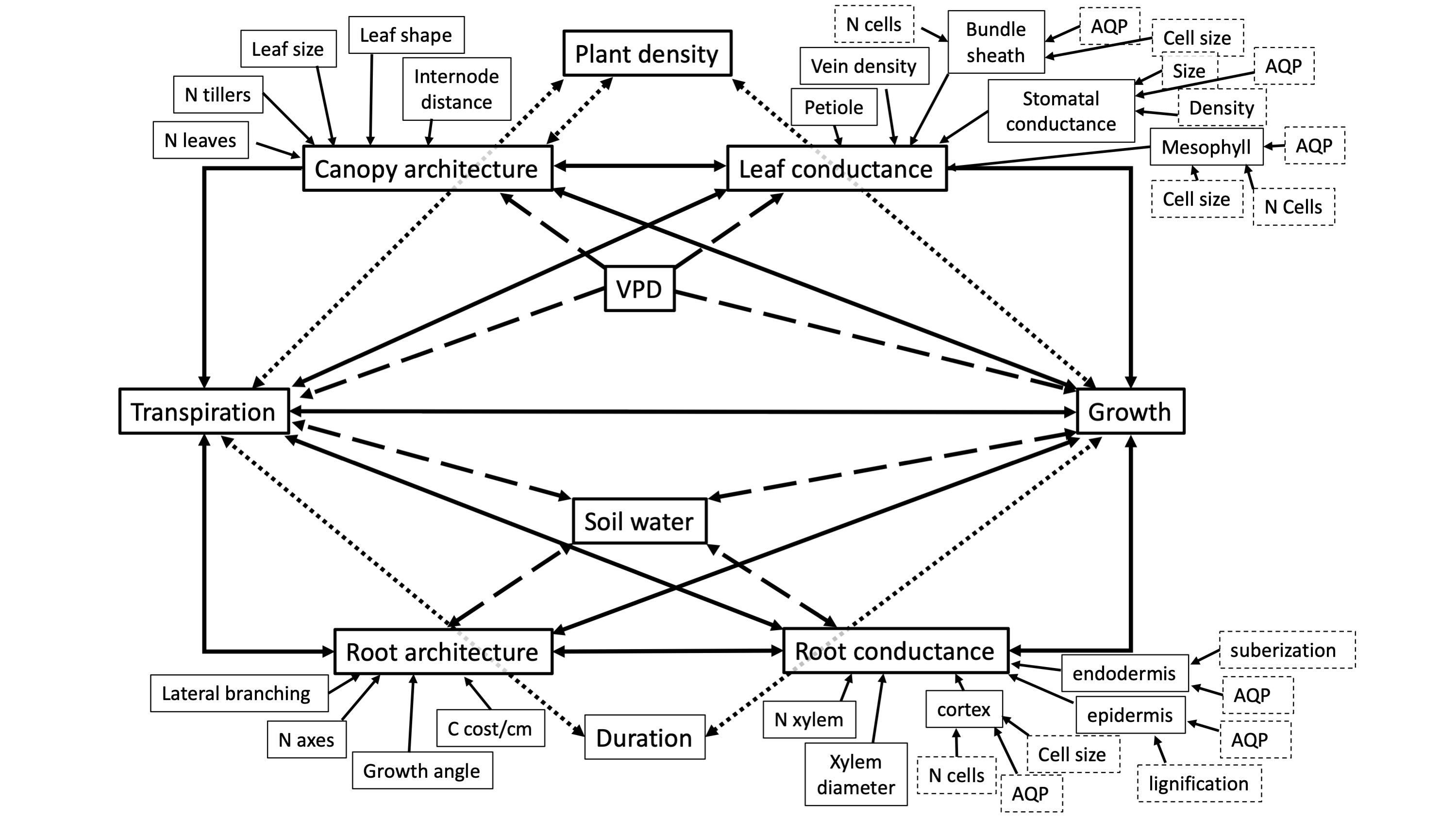

Supplement: Supplementary Figure 1 — Functional projection of plant hydraulic regulation foregrounding nested structural hierarchies associated with the four general mechanisms (root and shoot architecture and conductance) governing transpiration and growth. Solid arrows depict biochemical and hydraulic signaling mechanisms within and between structural hierarchies and include transmembrane pressure potential, xylem pressure potential, pH, hormones, as well as carbohydrate, and nutrient concentration. Dashed arrows indicate interactions with environmental factors. Dotted arrows indicate interactions with phenology and planting density. [file Figure_3.JPEG]
