# Supplementary material for: Optimizing Crop Water Use for Drought and Climate Change Adaptation Requires a Multi-Scale Approach
Source: Front Plant Sci. 2022 Apr 29;13:824720. doi: 10.3389/fpls.2022.824720 (PMC9100818; doi:10.3389/fpls.2022.824720)
Supplement: Supplementary file 2 [file Table_2.DOCX]

Supplemental Table 1. List of references and reference numbers for Table 1

Number Reference

1 (Henry et al., 2019)

2 (Bao et al., 2014)

3 (DIetrich et al., 2017)

4 (Sakurai-Ishikawa et al., 2011)

5 (Reddy et al., 2017)

6 (Medina et al., 2019)

7 (Nada and Abogadallah, 2014)

8 (Pou et al., 2013)

9 (Li et al., 2008)

10 (Ehlert et al., 2009)

11 (Ding et al., 2020)

12 (Barberon et al., 2016)

13 (Buckley et al., 2015)

14 (Sinclair et al., 2008)

15 (Grondin et al., 2016)

16 (Sutka et al., 2011)

17 (Meng et al., 2016)

18 (Ye et al., 2020)

19 (Kelly et al., 2014)

20 (Cui et al., 2008)J

21 (Choudhary and Sinclair, 2014)

23 (Ranathunge et al., 2004)

24 (Bramley et al., 2009)

25 (Parent et al., 2009)

26 (Zhou et al., 2012)

27 (Prado et al., 2019)

28 (Sadok and Sinclair, 2010)

29 (Shatil-Cohen et al., 2011)

30 (Prado et al., 2013)

31 (Ding et al., 2019)

32 (Nada and Abogadallah, 2020)

33 (Ding et al., 2018)

34 (Di Pietro et al., 2013)

35 (Sade et al., 2015)

36 (Sade et al., 2014)

37 (Zwieniecki et al., 2007)

38 (Postaire et al., 2010)

39 (Javot et al., 2003)

40 (Robbins and Dinneny, 2018)

41 (Henry et al., 2012)

42 (Sack and Holbrook, 2006)

43 (Kholová et al., 2010b)

44 (Tharanya et al., 2018)

45 (Kholová et al., 2016)

46 (Karthika et al., 2019)

47 (Aparna et al., 2015)

48 (Sivasakthi et al., 2020)

49 (Choudhary et al., 2020)

50 (Riar et al., 2015)

51 (Shekoofa et al., 2016)

52 (Choudhary et al., 2013)

53 (George-Jaeggli et al., 2017)

54 (Carminati and Javaux, 2020)

55 (Javaux and Carminati, 2021)

56 (Kholová et al., 2010a)

**References for Supplemental Table 1.**

Aparna, K., Nepolean, T., Srivastsava, R. K., Kholová, J., Rajaram, V., Kumar, S., et al. (2015). Quantitative trait loci associated with constitutive traits control water use in pearl millet [Pennisetum glaucum (L.) R. Br.]. *Plant Biol.* 17, 1073–1084. doi:10.1111/PLB.12343.

Bao, Y., Aggarwal, P., Robbins, N. E., Sturrock, C. J., Thompson, M. C., Tan, H. Q., et al. (2014). Plant roots use a patterning mechanism to position lateral root branches toward available water. *Proc. Natl. Acad. Sci. U. S. A.* 111, 9319–9324. doi:10.1073/pnas.1400966111.

Barberon, M., Vermeer, J. E. M., De Bellis, D., Wang, P., Naseer, S., Andersen, T. G., et al. (2016). Adaptation of Root Function by Nutrient-Induced Plasticity of Endodermal Differentiation. *Cell* 164, 447–459. doi:10.1016/j.cell.2015.12.021.

Bramley, H., Turner, N. C., Turner, D. W., and Tyerman, S. D. (2009). Roles of morphology, anatomy, and aquaporins in determining contrasting hydraulic behavior of roots. *Plant Physiol.* 150, 348–364. doi:10.1104/pp.108.134098.

Buckley, T. N., John, G. P., Scoffoni, C., and Sack, L. (2015). How does leaf anatomy influence water transport outside the xylem? *Plant Physiol.* 168, 1616–1635. doi:10.1104/pp.15.00731.

Carminati, A., and Javaux, M. (2020). Soil Rather Than Xylem Vulnerability Controls Stomatal Response to Drought. *Trends Plant Sci.* 25, 868–880. doi:10.1016/j.tplants.2020.04.003.

Choudhary, S., Guha, A., Kholova, J., Pandravada, A., Messina, C. D., Cooper, M., et al. (2020). Maize, sorghum, and pearl millet have highly contrasting species strategies to adapt to water stress and climate change-like conditions. *Plant Sci.* 295, 110297. doi:10.1016/j.plantsci.2019.110297.

Choudhary, S., Mutava, R. N., Shekoofa, A., Sinclair, T. R., and Prasad, P. V. V. (2013). Is the stay-green trait in sorghum a result of transpiration sensitivity to either soil drying or vapor pressure deficit? *Crop Sci.* 53, 2129–2134. doi:10.2135/cropsci2013.01.0043.

Choudhary, S., and Sinclair, T. R. (2014). Hydraulic conductance differences among sorghum genotypes to explain variation in restricted transpiration rates. *Funct. Plant Biol.* 41, 270–275. doi:10.1071/FP13246.

Cui, X. H., Hao, F. S., Chen, H., Chen, J., and Wang, X. C. (2008). Expression of the Vicia faba VfPIP1 gene in Arabidopsis thaliana plants improves their drought resistance. *J. Plant Res.* 121, 207–214. doi:10.1007/s10265-007-0130-z.

Di Pietro, M., Vialaret, J., Hem, S., Prado, K., Rossignol, M., Maurel, C., et al. (2013). Coordinated post-translational responses of aquaporins to abiotic and nutritional stimuli in arabidopsis roots. *Mol. Cell. Proteomics* 12, 3886–3897. doi:10.1074/mcp.M113.028241.

DIetrich, D., Pang, L., Kobayashi, A., Fozard, J. A., Boudolf, V., Bhosale, R., et al. (2017). Root hydrotropism is controlled via a cortex-specific growth mechanism. *Nat. Plants* 3. doi:10.1038/nplants.2017.57.

Ding, L., Li, Y., Gao, L., Lu, Z., Wang, M., Ling, N., et al. (2018). Aquaporin expression and water transport pathways inside leaves are affected by nitrogen supply through transpiration in rice plants. *Int. J. Mol. Sci.* 19. doi:10.3390/ijms19010256.

Ding, L., Milhiet, T., Couvreur, V., Nelissen, H., Meziane, A., Parent, B., et al. (2020). Modification of the expression of the aquaporin ZmPIP2;5 affects water relations and plant growth. *Plant Physiol.* 182, 2154–2165. doi:10.1104/PP.19.01183.

Ding, L., Uehlein, N., Kaldenhoff, R., Guo, S., Zhu, Y., and Kai, L. (2019). Aquaporin PIP2;1 affects water transport and root growth in rice (Oryza sativa L.). *Plant Physiol. Biochem.* 139, 152–160. doi:10.1016/j.plaphy.2019.03.017.

Ehlert, C., Maurel, C., Tardieu, F., and Simonneau, T. (2009). Aquaporin-mediated reduction in maize root hydraulic conductivity impacts cell turgor and leaf elongation even without changing transpiration. *Plant Physiol.* 150, 1093–1104. doi:10.1104/pp.108.131458.

George-Jaeggli, B., Mortlock, M. Y., and Borrell, A. K. (2017). Bigger is not always better: Reducing leaf area helps stay-green sorghum use soil water more slowly. *Environ. Exp. Bot.* 138, 119–129. doi:10.1016/j.envexpbot.2017.03.002.

Grondin, A., Mauleon, R., Vadez, V., and Henry, A. (2016). Root aquaporins contribute to whole plant water fluxes under drought stress in rice (Oryza sativa L.). *Plant Cell Environ.* 39, 347–365. doi:10.1111/pce.12616.

Henry, A., Cal, A. J., Batoto, T. C., Torres, R. O., and Serraj, R. (2012). Root attributes affecting water uptake of rice (Orza sativa) under drought. *J. Exp. Bot.* 63, 4751–4763. doi:10.1093/jxb/ers150.

Henry, A., Stuart-Williams, H., Dixit, S., Kumar, A., and Farquhar, G. (2019). Stomatal conductance responses to evaporative demand conferred by rice drought-yield quantitative trait locus qDTY12.1. *Funct. Plant Biol.* 46, 660–669. doi:10.1071/FP18126.

Javaux, M., and Carminati, A. (2021). Soil hydraulics affect the degree of isohydricity. *Plant Physiol.* 186, 1378–1381. doi:10.1093/PLPHYS/KIAB154.

Javot, H., Lauvergeat, V., Santoni, V., Martin-Laurent, F., Güçlü, J., Vinh, J., et al. (2003). Role of a single aquaporin isoform in root water uptake. *Plant Cell* 15, 509–522. doi:10.1105/tpc.008888.

Karthika, G., Kholova, J., Alimagham, S., Ganesan, M., Chadalavada, K., Kumari, R., et al. (2019). Measurement of transpiration restriction under high vapor pressure deficit for sorghum mapping population parents. *Plant Physiol. Reports* 24, 74–85. doi:10.1007/s40502-019-0432-x.

Kelly, G., Sade, N., Attia, Z., Secchi, F., Zwieniecki, M., Holbrook, N. M., et al. (2014). Relationship between hexokinase and the aquaporin PIP1 in the regulation of photosynthesis and plant growth. *PLoS One* 9. doi:10.1371/journal.pone.0087888.

Kholová, J., Hash, C. T., Kakkera, A., Koová, M., and Vadez, V. (2010a). Constitutive water-conserving mechanisms are correlated with the terminal drought tolerance of pearl millet [Pennisetum glaucum (L.) R. Br.]. *J. Exp. Bot.* 61, 369–377. doi:10.1093/jxb/erp314.

Kholová, J., Hash, C. T., Kumar, P. L., Yadav, R. S., Koová, M., and Vadez, V. (2010b). Terminal drought-tolerant pearl millet [Pennisetum glaucum (L.) R. Br.] have high leaf ABA and limit transpiration at high vapour pressure deficit. *J. Exp. Bot.* 61, 1431–1440. doi:10.1093/jxb/erq013.

Kholová, J., Zindy, P., Malayee, S., Baddam, R., Murugesan, T., Kaliamoorthy, S., et al. (2016). Component traits of plant water use are modulated by vapour pressure deficit in pearl millet (Pennisetum glaucum (L.) R.Br.). *Funct. Plant Biol.* 43, 423–437. doi:10.1071/FP15115.

Li, G. W., Peng, Y. H., Yu, X., Zhang, M. H., Cai, W. M., Sun, W. N., et al. (2008). Transport functions and expression analysis of vacuolar membrane aquaporins in response to various stresses in rice. *J. Plant Physiol.* 165, 1879–1888. doi:10.1016/j.jplph.2008.05.002.

Medina, S., Vicente, R., Nieto-Taladriz, M. T., Aparicio, N., Chairi, F., Vergara-Diaz, O., et al. (2019). The plant-transpiration response to vapor pressure deficit (VPD) in durum wheat is associated with differential yield performance and specific expression of genes involved in primary metabolism and water transport. *Front. Plant Sci.* 9, 1–19. doi:10.3389/fpls.2018.01994.

Meng, D., Walsh, M., and Fricke, W. (2016). Rapid changes in root hydraulic conductivity and aquaporin expression in rice (Oryza sativa l.) in response to shoot removal – Xylem tension as a possible signal. *Ann. Bot.* 118, 809–819. doi:10.1093/aob/mcw150.

Nada, R. M., and Abogadallah, G. M. (2014). Aquaporins are major determinants of water use efficiency of rice plants in the field. *Plant Sci.* 227, 165–180. doi:10.1016/j.plantsci.2014.08.006.

Nada, R. M., and Abogadallah, G. M. (2020). Contrasting root traits and native regulation of aquaporin differentially determine the outcome of overexpressing a single aquaporin (OsPIP2;4) in two rice cultivars. *Protoplasma* 257, 583–595. doi:10.1007/s00709-019-01468-x.

Parent, B., Hachez, C., Redondo, E., Simonneau, T., Chaumont, F., and Tardieu, F. (2009). Drought and abscisic acid effects on aquaporin content translate into changes in hydraulic conductivity and leaf growth rate: A trans-scale approach1[w][OA]. *Plant Physiol.* 149, 2000–2012. doi:10.1104/pp.108.130682.

Postaire, O., Tournaire-Roux, C., Grondin, A., Boursiac, Y., Morillon, R., Schäffner, A. R., et al. (2010). A PIP1 aquaporin contributes to hydrostatic pressure-induced water transport in both the root and rosette of Arabidopsis. *Plant Physiol.* 152, 1418–1430. doi:10.1104/pp.109.145326.

Pou, A., Medrano, H., Flexas, J., and Tyerman, S. D. (2013). A putative role for TIP and PIP aquaporins in dynamics of leaf hydraulic and stomatal conductances in grapevine under water stress and re-watering. *Plant, Cell Environ.* 36, 828–843. doi:10.1111/pce.12019.

Prado, K., Boursiac, Y., Tournaire-Roux, C., Monneuse, J. M., Postaire, O., Da Ines, O., et al. (2013). Regulation of Arabidopsis leaf hydraulics involves light-dependent phosphorylation of aquaporins in veins. *Plant Cell* 25, 1029–1039. doi:10.1105/tpc.112.108456.

Prado, K., Cotelle, V., Li, G., Bellati, J., Tang, N., Tournaire-Roux, C., et al. (2019). Oscillating aquaporin phosphorylation and 14-3-3 proteins mediate the circadian regulation of leaf hydraulics. *Plant Cell* 31, 417–429. doi:10.1105/tpc.18.00804.

Ranathunge, K., Kotula, L., Steudle, E., and Lafitte, R. (2004). Water permeability and reflection coefficient of the outer part of young rice roots are differently affected by closure of water channels (aquaporins) or blockage of apoplastic pores. *J. Exp. Bot.* 55, 433–447. doi:10.1093/jxb/erh041.

Reddy, P. S., Tharanya, M., Sivasakthi, K., Srikanth, M., Hash, C. T., Kholova, J., et al. (2017). Molecular cloning and expression analysis of Aquaporin genes in pearl millet [Pennisetum glaucum (L) R. Br.] genotypes contrasting in their transpiration response to high vapour pressure deficits. *Plant Sci.* 265, 167–176. doi:10.1016/j.plantsci.2017.10.005.

Riar, M. K., Sinclair, T. R., and Prasad, P. V. V. (2015). Persistence of limited-transpiration-rate trait in sorghum at high temperature. *Environ. Exp. Bot.* 115, 58–62. doi:10.1016/j.envexpbot.2015.02.007.

Robbins, N. E., and Dinneny, J. R. (2018). Growth is required for perception of water availability to pattern root branches in plants. *Proc. Natl. Acad. Sci. U. S. A.* 115, E822–E831. doi:10.1073/pnas.1710709115.

Sack, L., and Holbrook, N. M. (2006). Leaf Hydraulics. *Annu. Rev. Plant Biol.* 57, 361–381. doi:10.1146/annurev.arplant.56.032604.144141.

Sade, N., Shatil-Cohen, A., Attia, Z., Maurel, C., Boursiac, Y., Kelly, G., et al. (2014). The Role of Plasma Membrane Aquaporins in Regulating the Bundle Sheath-Mesophyll Continuum and Leaf Hydraulics. *Plant Physiol.* 166, 1609–1620. doi:10.1104/pp.114.248633.

Sade, N., Shatil-Cohen, A., and Moshelion, M. (2015). Bundle-sheath aquaporins play a role in controlling Arabidopsis leaf hydraulic conductivity. *Plant Signal. Behav.* 10, 1–4. doi:10.1080/15592324.2015.1017177.

Sadok, W., and Sinclair, T. R. (2010). Transpiration response of “slow-wilting” and commercial soybean (Glycine max (L.) Merr.) genotypes to three aquaporin inhibitors. *J. Exp. Bot.* 61, 821–829. doi:10.1093/jxb/erp350.

Sakurai-Ishikawa, J., Murai-Hatano, M., Hayashi, H., Ahamed, A., Fukushi, K., Matsumoto, T., et al. (2011). Transpiration from shoots triggers diurnal changes in root aquaporin expression. *Plant, Cell Environ.* 34, 1150–1163. doi:10.1111/j.1365-3040.2011.02313.x.

Shatil-Cohen, A., Attia, Z., and Moshelion, M. (2011). Bundle-sheath cell regulation of xylem-mesophyll water transport via aquaporins under drought stress: A target of xylem-borne ABA? *Plant J.* 67, 72–80. doi:10.1111/j.1365-313X.2011.04576.x.

Shekoofa, A., Sinclair, T. R., Messina, C. D., and Cooper, M. (2016). Variation among maize hybrids in response to high vapor pressure deficit at high temperatures. *Crop Sci.* 56, 392–396. doi:10.2135/cropsci2015.02.0134.

Sinclair, T. R., Zwieniecki, M. A., and Holbrook, N. M. (2008). Low leaf hydraulic conductance associated with drought tolerance in soybean. *Physiol. Plant.* 132, 446–451. doi:10.1111/j.1399-3054.2007.01028.x.

Sivasakthi, K., Tharanya, M., Zaman-Allah, M., Kholová, J., Thirunalasundari, T., and Vadez, V. (2020). Transpiration difference under high evaporative demand in chickpea (Cicer arietinum L.) may be explained by differences in the water transport pathway in the root cylinder. *Plant Biol.* 22, 769–780. doi:10.1111/plb.13147.

Sutka, M., Li, G., Boudet, J., Boursiac, Y., Doumas, P., and Maurel, C. (2011). Natural variation of root hydraulics in Arabidopsis grown in normal and salt-stressed conditions. *Plant Physiol.* 155, 1264–1276. doi:10.1104/pp.110.163113.

Tharanya, M., Sivasakthi, K., Barzana, G., Kholová, J., Thirunalasundari, T., and Vadez, V. (2018). Pearl millet (Pennisetum glaucum) contrasting for the transpiration response to vapour pressure deficit also differ in their dependence on the symplastic and apoplastic water transport pathways. *Funct. Plant Biol.* 45, 719–736. doi:10.1071/FP17161.

Ye, H., Song, L., Schapaugh, W. T., Ali, M. L., Sinclair, T. R., Riar, M. K., et al. (2020). The importance of slow canopy wilting in drought tolerance in soybean. *J. Exp. Bot.* 71, 642–652. doi:10.1093/jxb/erz150.

Zhou, S., Hu, W., Deng, X., Ma, Z., Chen, L., Huang, C., et al. (2012). Overexpression of the Wheat Aquaporin Gene, TaAQP7, Enhances Drought Tolerance in Transgenic Tobacco. *PLoS One* 7. doi:10.1371/journal.pone.0052439.

Zwieniecki, M. A., Brodribb, T. J., and Holbrook, N. M. (2007). Hydraulic design of leaves: Insights from rehydration kinetics. *Plant, Cell Environ.* 30, 910–921. doi:10.1111/j.1365-3040.2007.001681.x.
